# Maize-Derived Lactic Acid Bacteria with Probiotic Traits and Antifungal Activity: Candidate Functional Starter Cultures and Bio-Preservatives

**DOI:** 10.3390/foods15020209

**Published:** 2026-01-07

**Authors:** Adeola O. Aasa, Samkelo Malgas, Mapitsi Silvester Thantsha

**Affiliations:** Department of Biochemistry, Genetics and Microbiology, Faculty of Natural and Agricultural Sciences, University of Pretoria, Hatfield 0028, South Africa; u16397518@tuks.co.za (A.O.A.); samkelo.malgas@up.ac.za (S.M.)

**Keywords:** *Alternaria*, antifungal, *Fusarium*, maize, lactic acid bacteria, probiotics

## Abstract

Contamination of agricultural products such as maize by fungi is a significant concern worldwide, as it can compromise food safety and quality. In recent years, the use of microorganisms as natural food preservatives has gained interest. Probiotic lactic acid bacteria (LAB) and their metabolites are considered a promising strategy to reduce fungal growth and limit other food contaminants. This study aimed to characterize, screen and compare the probiotic properties and antifungal activity of LAB of maize origin. A total of 23 LAB isolates obtained from untreated maize grains were identified through 16S rRNA gene sequencing as *Weissella viridenscens* (34.7%), *Pediococcus pentosaceus* (34.7%), *Enterococcus durans* (17.4%), *Leuconostoc citreum* (9%), and *Enterococcus faecium* (4.3%). All isolates demonstrated acid, phenol, and bile salt tolerance; surface hydrophobicity; and antagonistic activity against selected bacterial foodborne pathogens. Notably, *Enterococcus* sp. showed the strongest inhibitory activity against *Escherichia coli* ATCC 5211 (21 mm inhibition zone) and *Staphylococcus aureus* (17 mm inhibition zone), whereas *Pediococcus* sp. exhibited the highest antagonistic effect against *Listeria monocytogenes* (18.7 mm inhibition zone). Furthermore, *E. durans* and *P. pentosaceus* demonstrated the strongest antifungal activity, effectively inhibiting the growth of *Alternaria tenuissima* (F22FR) and *Fusarium oxysporum* (F44FR), respectively. Overall, all the LAB strains isolated from this study showed considerable potential for use in the food industry as probiotics, starter cultures for functional food fermentations, bio-preservatives and biocontrol agents against toxigenic fungi and pathogenic bacteria, with *E. durans* standing out for its exceptional performance. Future research will explore the ability of these isolates and/or their enzymes to degrade mycotoxins commonly found in maize, a staple food in many African countries.

## 1. Introduction

Maize is one of the major cereal crops cultivated and produced globally [1]. It is an essential meal, particularly in Sub-Saharan Africa. It is a staple diet for many South Africans and a substantial component of animal feed. However, it can become infected with phytopathogenic and toxigenic fungi, causing diseases in the vegetative and reproductive organs of the plant, which can result in reduced yields [2,3]. In addition to these diseases, some fungi can produce mycotoxins and deposit them in damaged plant tissues, which may pose a considerable risk to humans and animals when such affected maize enters the food and feed chains. Various chemical and physical approaches have been employed to mitigate the impact of this menace on sustainable food safety and production. These approaches exhibit several limitations, such as nutritional loss, reduced effectiveness, safety issues, and the high costs associated with the required facilities. The limited availability of sustainable and environmentally friendly methods for combating fungi and their mycotoxins in food products has led to the investigation of biologically safe alternatives [4]. Biological detoxification, which includes microbial and enzyme applications, is environmentally friendly as it is effective, irreversible, and does not produce toxic by-products [5]. Several microorganisms, particularly probiotics, have been reported to have the ability to inhibit fungal growth and mycotoxin production.

Probiotic strains are popularly used as supplements due to their positive impact on human and animal well-being. They can be isolated from various sources, including humans, animals, and plants [6]. Probiotic qualities are strain-specific; hence, in order to discover novel and more dependable strains, every new prospective probiotic strain must be well-identified and screened. Lactic acid bacteria (LAB) and Bifidobacteria have been the most thoroughly investigated probiotics for decades [7]. LAB have been extensively used as probiotics because of their GRAS (Generally Regarded as Safe) status endorsed by the FDA and granted QPS (Qualified Presumption of Safety) status by the European Food Safety Authority (EFSA) [8].

LAB strains from the genera *Lactobacillus*, *Enterococcus*, *Lactococcus*, *Pediococcus*, *Weissella*, *Leuconostoc*, and *Streptococcus* are common probiotics and can be found naturally on grain surfaces and in the environment [9]. Furthermore, LAB are also known for producing antibacterial compounds that are useful in food and feed preservation. As a result, they are frequently used in the food industry due to the increased demand for reducing chemical usage in this industry.

LAB can produce antimicrobial or antifungal compounds that can serve as alternatives to chemical preservatives to overcome food-borne pathogens [10]. These compounds include bacteriocins, organic acids, primary alcohols, fatty acids, 3-hydroxy decanoic acid, carboxylic acids, amines, amino acids, 5-oxododecanoic acid, phenylpropanoids, pyrimidines, and hydrogen peroxide [10,11,12,13,14]. The capacity of LAB to inhibit growth of food pathogens in food matrices is dependent on their production of these metabolites.

Foods containing probiotics, often known as functional foods, provide a variety of therapeutic benefits, including antihypertensive, anticancer, hypoglycemic, antioxidant, and immunomodulatory properties [15,16]. LAB are important in alleviating gastrointestinal (GI) infections such as dysentery, respiratory tract infections, typhoid, and diarrhoea [17]. They are used in food and feed industries, and these industries are constantly seeking novel strains with potential to enhance sensory and product quality [3]. Therefore, investigating new LAB identified from unexplored niches may lead to the isolation of unique strains with promising biocontrol attributes, plant growth stimulation effects, capacity to enhance nutritional content of food, and inhibitory effects against phytopathogenic microbes [18,19]. Overall, finding novel probiotic strains with high survival rates in food processing stages and high resistance to GIT conditions due to mechanisms such as pH homeostasis, restriction of proton permeation, and enhancement of proton pumps is of great importance to delivering their beneficial effects [20]. The present study aimed to compare the probiotic properties and antifungal activity of different LAB isolates of maize grain origin.

## 2. Materials and Methods

Three different untreated white maize samples allocated specific identifier codes, DKC-75-45, DKC-75-65, and DKC-77-77, were randomly collected from the storage facility of the Department of Plant Sciences, University of Pretoria, and one sample was purchased from a local market in Hammanskral, Pretoria (Latitude: −25°23′59.99″, S, and longitude: 28°16′60.00″). The samples were transferred to the Probiotic and Food Technology Laboratory at the Department of Biochemistry, Genetics, and Microbiology, University of Pretoria, where they were kept at room temperature until use.

### 2.1. Isolation, Identification, and Characterisation of Lactic Acid Bacteria

The maize samples were pulverized using a stomacher 400 (Seward, WestSussex, UK) for 20 min and then 5 g of milled maize was soaked in a Duran bottle containing 50 mL of autoclaved deionized water for three days at room temperature. After three days, the suspensions were serially diluted until 1:6 and then 100 µL aliquots of each dilution were pour-plated in triplicate onto MRS agar supplemented with cycloheximide (0.1 g/L). The plates were incubated at 37 °C in anaerobic jars in the presence of Anaerocult A gaspak for 48 h [21]. The individual colonies with various morphologies were randomly selected from the agar plates at the highest dilution. Identification of the isolates at the genus level was carried out using phenotypic characterisation, Gram staining, and the catalase test [22]. Gram-positive, non-motile, catalase-negative cocci and rods were cultured anaerobically in MRS broth and incubated using SHELLAB incubator (SHELDON INC, Cornelius, OR, USA) for 24 h at 37 °C without shaking. Subsequently, the broth culture was plated onto MRS agar and incubated for 48 h at 37 °C.

For molecular characterisation, the total genomic DNA of isolates was extracted using the *Quick*-DNA^TM^ Fungal/Bacteria Miniprep Kit (Zymo Research Corp., Irvine, CA, USA) according to the manufacturer’s instructions. Polymerase chain reaction (PCR) was carried out using the Phusion High-Fidelity PCR Kit (New England Biolabs, Ipswich, MA, USA) following the manufacturer’s protocol. Amplification of 16S rDNA was performed using both primers 27F [5′-AGAGTTTGATCCTGGCTCAG-3″] and 1492R [5′-TAGGACTTAACCCCAATCGC-3″] [23]. The thermal cycling was carried out using the initial denaturation of 98 °C for 3 min, followed by 30 cycles of DNA amplification. Each cycle consists of denaturation at 98 °C for 30 s, annealing at 58 °C for 30 s and extension at 68 °C for 1 min. The final extension occurred at 68 °C for 5 min. The amplified fragments were separated on 1% agarose gel stained with ethidium bromide, visualized using a Gel Doc ^TM^ EZ imaging system with Image Lab software (version 6) (Bio-Rad Laboratories, Inc., Irvine, CA, USA).

For sequence analysis of the 16S rDNA, the PCR products were purified with the QIAquick^®^ PCR purification kit (Qiagen, QiagenStr 1, Hilden, Germany) according to the manufacturer’s instructions. The purified products were sequenced separately with both 27F and 1492R primers. The sequencing reaction mixture contained 5.75 µL of purified DNA, 1 µL of sequencing buffer, 1.25 µL of primer (1.0 µmol), and 2 µL of Big Dye terminator. The sequencing was performed using the Big-Dye sequencer ABIPRISM 313 × 1. Sequence homologies were examined by comparing the obtained sequences with available sequences in the GenBank database using the NCBI BLAST program (blastn) (https://blast.ncbi.nlm.nih.gov/Blast.cgi (accessed on 13 November 2023)).

### 2.2. Isolation, Identification, and Characterization of Toxigenic Fungi

The fungi used for the antifungal activity analysis of the maize-derived LAB isolates also originated from the same maize samples. The maize grains were milled, as was done for LAB isolation. The maize suspension was plated onto potato dextrose agar and Rose Bengal chloramphenicol agar plates, which were subsequently incubated at 28 °C for 3–4 days. Phenotypic characterization and microscopic identification of the fungi were done using the method described by Aasa et al. [24]. For molecular characterization, the internal transcribed spacer (ITS) region was amplified using ITS1 (5′-TCC-GTA-GGT-GAA-CCT-GCG-G-3′ (forward) and ITS4 (5′-TCC-TCC-GCT-TAT-TGA-TAT-GC-3′ (reverse) primers. The PCR and sequencing were performed as described above. The acquired sequences were compared to known sequences in GenBank using BLAST software.

### 2.3. Screening of LAB for Probiotic Attributes

#### 2.3.1. Acid Tolerance Assay

The acid tolerance assay was performed as described by Padmavathi et al. [25] with minor modifications. The LAB isolates were cultured overnight in MRS broth, and the concentration of the culture was adjusted to 0.3 optical density (OD_600_). Then 1 mL of the culture was inoculated into 9 mL of phosphate buffer solution (PBS) adjusted to different pH levels (1.5, 2, 2.5, and 3) with HCL (5M) (Merck, Germany) and incubated at 37 °C for 3 h. The viability of the culture was determined at 0 and 3 h by plating it onto MRS agar plates. Cultures grown in MRS broth (pH 7) were used as positive controls. The experiment was conducted in three independent trials. The percentage growth of the culture was calculated using the equation:

$$\%\;\mathrm{Growth}\;=\;\frac{A1-A0}{A0}\times 100$$

where *A*0 = Absorbance incubation (0 h) and *A*1 = absorbance after incubation.

#### 2.3.2. Phenol Tolerance Assay

To evaluate the in vitro phenol tolerance of the LAB strains, three phenol concentrations (0.1, 0.2, and 0.4% *w*/*v*) were used for assessment following the method described by Shehata et al. [26]. Each MRS tube with a specific phenol concentration was inoculated with 1% (*v*/*v*) of overnight-grown culture and incubated for 24 h at 37 °C. LAB strains cultured in MRS broth were used as a positive control. The viability of LAB strains was evaluated after incubation using a Thermo Scientific Multiskan GO spectrophotometer (Thermofisher, Walthan, MA, USA) at 600 nm. The analysis was done in duplicate.

#### 2.3.3. Bile Salt Tolerance Assay

The capacity of the LAB to grow in the presence of bile salts was determined using a method described by Shehata et al. [26]. Each LAB culture (10^8^ CFU/mL) was inoculated into MRS media enriched with 0.3% or 0.5% *w*/*v* bile salt (Bio-Lab). Cultures grown in MRS broth without bile salts were used as positive control. The cultures were incubated for 24 h at 37 °C, then subsamples were withdrawn at 0, 3, 6, 18, and 24 h for viable count determination.

#### 2.3.4. Antibiotic Susceptibility Test

The antibiotic susceptibility of the LAB cultures was examined using the disc diffusion technique as described by Makete et al. [23]. Briefly, the MRS-cysHCL broth culture of each isolate was adjusted to 1 × 10^8^ CFU/mL, and then 100 µL was spread plated onto the MRS agar plate. Then an antibiotic disc ring (mastrings) with the following antibiotics: chloramphenicol (25 µg), erythromycin (5 µg), fusidic acid (10 µg), oxacillin (5 µg), novobiocin (5 µg), penicillin G (1 unit), streptomycin (10 µg), and tetracycline (25 µg), was laid onto the agar plate, and the plates were anaerobically incubated for 24 h at 37 °C. The sizes of the inhibitory zones were measured, averaged, and categorised across three readings (Table 1). Classification of the diameters of the inhibitory zones followed the microbiological breakpoints described by CLSI [27] and Yerlikaya et al. [28] for chloramphenicol, erythromycin, tetracycline, streptomycin, penicillin G, oxacillin, novobiocin and fusidic acid as applied by Bouguerra et al. [29].

#### 2.3.5. Cell Surface Hydrophobicity

LAB adhesion to hydrocarbons was tested following the Srikham et al. [30] technique, with some modifications. Briefly, LAB strains were grown overnight in MRS broth, then centrifuged at 4000× *g* for 10 min at 4 °C. The cell pellets were collected and washed twice with 1× phosphate-buffered saline (PBS) (10 mM, pH 7.1), resuspended in the same buffer, and adjusted to an absorbance of 1.0 at 600 nm (A_0_). Bacterial suspensions (3 mL) were mixed separately with 1 mL of two solvents: toluene and chloroform (Merck, Germany). The tubes were vortexed for 30 s and then incubated for 30 min at room temperature. The aqueous phase was collected, and the absorbance at 600 nm (A_1_) was measured. The percentage cell surface hydrophobicity was calculated using the following formula:

$$CSH\;=\;1-\frac{A_{1}\;}{A_{0}}\times 100$$

where *CSH* = cell surface hydrophobicity, *A*_0_ = initial absorbance, and *A*_1_ = final absorbance.

#### 2.3.6. Antibacterial Activity Assay of Maize-Derived LAB Isolates

The agar well diffusion method described by Makete et al. [23] was used to test the inhibitory activity of the LAB against *Escherichia coli* ATCC 5211, *Listeria monocytogenes* ATCC 19,115 and *Staphylococcus aureus* ATCC 25923. The isolates were cultured in MRS broth (pH 6.5) for 48 h at 37 °C. The cultures were subsequently centrifuged with fixed-angle rotor (HERMLE Z-366K centrifuge, LABORTECHNIK, Wehingen, Germany) for 10 min at 2236× *g* at room temperature, and then the cell-free supernatants (CFS) were harvested. Overnight cultures of each pathogen were separately spread-plated onto Mueller–Hinton agar plates and then wells of 6 mm diameter were punctured onto the inoculated plates using a cork borer. Then 100 µL aliquot of the CFS (pH 6.5) of each LAB culture was dispensed into each well, and the plates were incubated at 37 °C for 24 h. The inhibition zones surrounding each well, from edge to zone border, were measured.

### 2.4. Screening for Antifungal Activity of Maize-Derived LAB Isolates

#### 2.4.1. Inhibition of Fungal Isolates by Viable LAB Cells

The LAB isolates were screened for antifungal activity using the agar-well diffusion technique [30]. The potato dextrose agar (PDA) plates containing 10^8^ CFU/mL of *Alternaria tenuissima* (F22FR) and *Fusarium oxysporum* (F44FR) conidia per mL were prepared, and 6 mm wells were made in the agar using a cork borer. One hundred microliters (100 µL) of LAB suspension (10^8^ CFU/mL) were added to the wells and allowed to diffuse into the solidified agar for an hour at room temperature before incubation at 28 °C for 48 h. The clear zones around the wells were measured and recorded.

#### 2.4.2. Inhibition of Fungi by Cell-Free Supernatants of LAB Cells

The LAB cultures were grown in MRS broth at 37 °C for 24 h. The cultures were centrifuged at 8000× *g* for 10 min, and the supernatants were collected and filtered through a 0.2 µm sterile filter (GVS Filter Technology, Findlay, OH, USA). The modified mould agar spot technique by Khalil et al. [31] was used for the assay. Two millilitres of the filter-sterilised supernatant were mixed with 8 mL of PDA at 55 °C and poured into the plates. For the fungal inoculum, the fungal mycelium of each test fungus was scraped from the PDA plates into the tube using a sterile scalpel, and 10 mL of PBS was added to the culture. The mixture was gently homogenised and left for 15 min to allow hyphae sedimentation. Ten microlitres of *Alternaria teinuissima* and *F. oxysporum* suspension were dropped at the centre of the agar plate and incubated for 4 days at 28 °C. The diameter of the mould was measured and then compared with the control, in which the supernatant was replaced with MRS broth. All tests were done in triplicate. The percentage of inhibition of radial mycelia growth by the CFS of LAB was calculated using the following formula:

$$I\;=\;\left(\frac{C-T}{C}\right)\times 100$$

where I = percentage of inhibition, C = diameter of the fungal colony in control, and T = diameter of the fungal colony in treated samples.

### 2.5. Statistical Analyses

Data obtained from this study were the mean of three replicates and expressed as mean ± standard deviation (SD) using SPSS version 28 (SPSS Inc., Chicago, IL, USA). For statistical comparisons, a one-way ANOVA with Turkey’s test was used. Data with *p* < 0.05 was considered statistically significant

## 3. Results

### 3.1. Identification of the Isolates

A total of 56 isolates were discovered in this study; among them, only 23 were Gram-positive, catalase-negative, non-spore-forming cocci and rods, which were selected as presumptive LAB (Table 2). The remaining 33 isolates that were either Gram-negative or Gram-positive but catalase-positive were discarded. After the preliminary phenotypic characterisation, the isolates were identified using the molecular characterisation of the 16S rDNA region.

Representative sequences from each genus and species were deposited in GenBank, and their assigned accession numbers are shown in Table 2. Most of the isolates showed a high degree of similarity with existing sequences in GenBank (Table 2). *E*. *durans* A05M (accession number OR794489.1) exhibited the highest level of similarity (100%) while the other isolates had similarities ranging from 90–99%, except for *P*. *pentosaceus* strains A02M and A017M, *W. viridescens* strains A035M, and A036M, which had similarities below 90% in GenBank.

**Figure 1:**
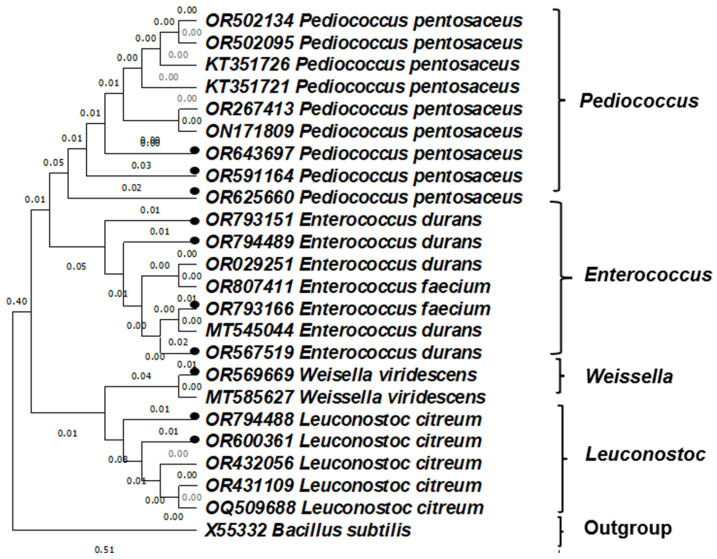
Phylogenetic tree based on partial 16S rRNA showing the relative position of LAB isolated from maize with GenBank representative. The isolates from this study are highlighted with black bullets. *Bacillus subtilis* was used as an outgroup.

A phylogenetic tree was created using isolates that had the specified accession number. The tree demonstrated the evolutionary relationships between the isolated LAB from this study and existing strains in GenBank. The isolates exhibit a close relationship with the established LAB on GenBank, as evidenced by the shorter branch lengths (0.00 to 0.05) between the isolated LAB and the related sequence from the GenBank (Figure 1).

### 3.2. Screening of Isolates for Potential Probiotics

#### 3.2.1. Acid Tolerance

**Figure 2:**
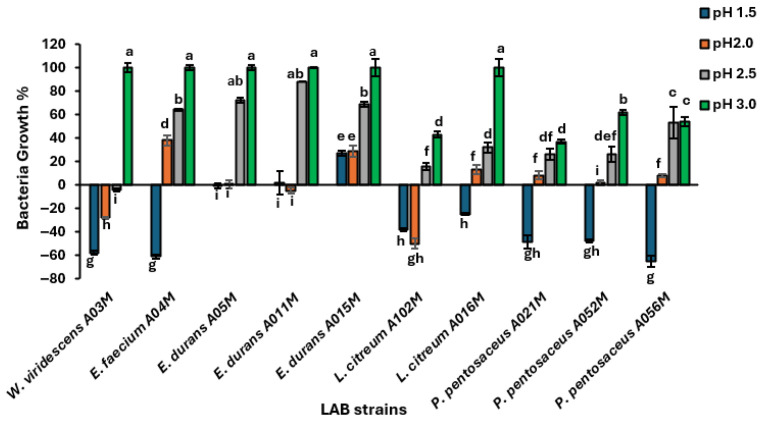
Acid tolerance of LAB isolates to pH 1.5, 2.0, 2.5 and 3.0, expressed as percentage growth relative to the initial absorbance (0 h). Negative values indicate reduced growth and probable loss of viability at extremely low pH. Each bar represents the mean of three independent readings; error bars indicate standard deviations. Bars with differing letters indicate significant differences between the strains (*p* < 0.05).

The ability to survive in the low pH of the gastrointestinal tract and arrive at the intended location in the proper physiological state is among the qualities that define an organism as a probiotic. Bacterial growth incubated at different pH levels (2.0, 2.5 and 3.0) was significantly different from that at pH 1.5 (*p* < 0.05). However, the bacteria growth at pH 3.0 was not significantly different among the *Weissella*, *Enterococcus* sp. and *L. citreum* A016M (*p* > 0.05). The statistically significant differences among isolates are indicated by different letters in Figure 2.

Most of the isolates could not survive at pH 1.5, except for the *Enterococcus* strains, which survived at pH 1.5, with *E. durans* strain A015M showing more acid tolerance than other *E. durans* strains (Figure 2). However, *Pediococcus*, *Leuconostoc*, and *Weissella* sp. are among the isolates that can withstand pH levels of 2.5 and 3. *Weissella viridescens* A03M, *E. durans* A011M, and *L. citreum* A102M lost viability at pH 2. Among the strains that tolerated pH 2, A04 showed the highest tolerance, followed closely by A015M. (Figure 2). Some isolates exhibited no growth or sparse growth as revealed by the absorbance of some isolates after incubation at pH 2.5 and pH 3 were low, which led to zero and/or negative percent when calculated.

All the isolates, except *W. viridescens*, survived at pH 2.5. The most tolerant at pH 2.5 was *E*. *durans* A011M. All the strains survived pH 3, with the survival of strains A03M, A04M, A05M, A011M, A015M, and A016M very similar. *P. pentosaceus* A021 was the least tolerant to pH 3. *Enterococcus durans* A015M showed more tolerance to acid than *E. faecium*, *L. citreum*, *W. viridescens*, and *P. pentosaceus.* Overall, *E. faecium* A04M and *E. durans* A015M were the most acid-tolerant, with the latter being the most acid-resistant.

#### 3.2.2. Phenol Tolerance

**Figure 3:**
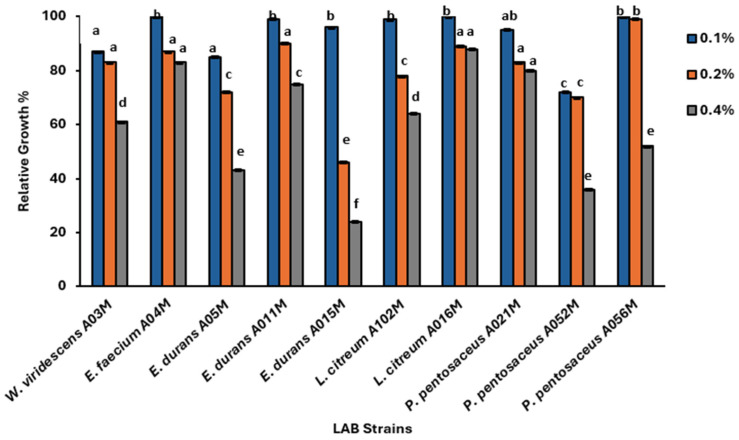
Tolerance of LAB to 0.1, 0.2 and 0.4% phenol. Each line represents the mean of two independent readings; error bars indicate standard deviations. Superscripts in lower case present the significant difference at *p* < 0.05 of each treatment across different strains.

The ability of the isolated LAB to survive in the presence of phenol was evaluated. The isolates were able to tolerate the various levels of phenol concentrations tested. It was observed that bacterial growth was inversely proportional to phenol concentration (Figure 3). The strains exhibited different degrees of sensitivity to the evaluated phenol concentrations; however, all the isolates exhibited ≥ 90% relative growth in 0.1% phenol, with significant differences (*p* < 0.05) across the trials (0.2 and 0.4%). In the presence of 0.2% phenol, only three strains (*E. durans* A015M, *L. citreum* A102M, and *P. pentosaceus* A052M) exhibited survival rate below 80%. At 0.4% phenol concentration, most of the isolates had their growth reduced to below 76%. Only *E. faecium* A04M, *L. citreum* A016M and *P. pentosaceus* A021 exhibited a relative growth of 80% at this phenol concentration.

#### 3.2.3. Bile Salt Tolerance

**Figure 4:**
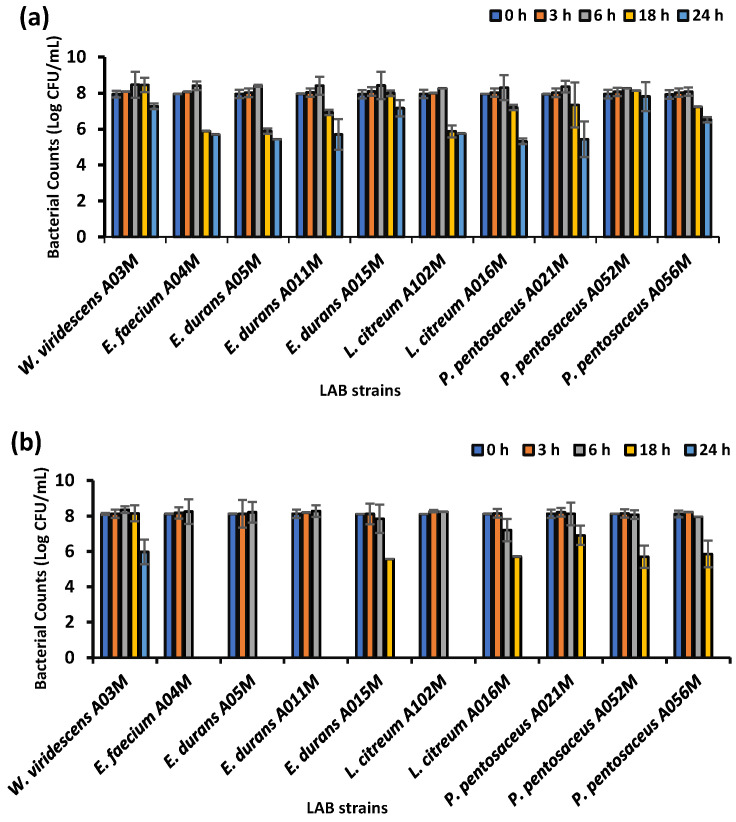
Bile tolerance of LAB isolates: (**a**) 0.3% and (**b**) 0.5% bile salts. Each line represents the mean of three readings; error bars indicate standard deviations.

The ability of the isolated LAB to survive in 0.3% and 0.5% bile salts was confirmed. All the LAB strains displayed good resistance to 0.3% and 0.5% bile salt for up to 6 h, as evidenced by survival and increased viable counts (Figure 4a,b). However, there was a decline in the number of viable cells after 6 h, ranging from 8.5 to 5.9 Log CFU/mL and 8.1 to 5.5 and even 0.0 Log CFU/mL in 0.3% and 0.5% bile salts, respectively. Only *P. pentosaceus* A052M, *E*. *durans* A015M, and *W*. *viridescens* A03M maintained viable counts of 7.00 ± 0.8 LogCFU/mL after exposure to 0.3% bile salt for up to 24 h (Figure 4a), making them the most bile-resistant strains. Overall viable counts were reduced from 8.09 to 5.32 LogCFU/mL after 24 h of exposure in 0.3. *Enteroccocus durans* A05M, *E. faecium* A04, and *E. durans* A011 were killed after 6 h of exposure to 0.5% bile salts. All the other strains survived for up to 18 h in this concentration of bile salts. *Weissella viridescens* was the only strain that survived after 24 h, with viable counts of 5.9 log CFU/mL (Figure 4b).

#### 3.2.4. Susceptibility of the Isolated LAB Strains to Antibiotics

All the LAB strains tested were susceptible to most of the antibiotics tested (Table 3 and Table 4). As shown in Table 3, all the isolates were susceptible or moderately susceptible to chloramphenicol, while 80% were susceptible to erythromycin and tetracycline, and all were resistant to streptomycin and oxacillin (Table 3). Only *E. faecium* A04M, *E. durans* A011M, and *L. citreum* A016M were sensitive to fusidic acid. *E. faecium* A04M, *E. durans* A015M, and *L. citreum* A016M were susceptible to novobiocin, but *W. viridescens* A03M, *P. pentosaceus* A052M, and *P. pentosaceus* A056M were penicillin G resistant. The number of bacterial strains susceptible and/or resistant to the antibiotics is presented in the bracket in Table 4.

#### 3.2.5. Cell Surface Hydrophobicity

**Figure 5:**
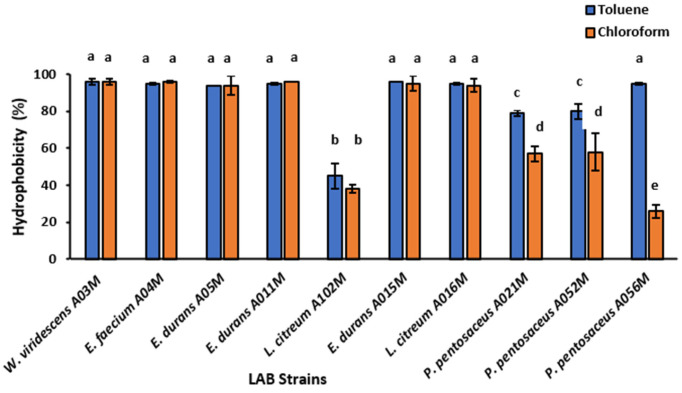
Surface hydrophobicity exhibited by the LAB isolates. Data are represented as the mean (n = 3); error bars indicate standard deviations. Superscripts in lower case present the significant difference at *p* < 0.05 between trials.

Figure 5 presents hydrophobicity of all the isolates to toluene and chloroform. The isolates exhibited high hydrophobicity (>80%) to toluene, except *L. citreum* A102M, which displayed less than 50% adhesion capability. All the *P. pentosaceus* strains exhibited the lowest hydrophobicity to chloroform. *L. citreum* A102M consistently showed low adhesion to both solvents. Overall, all the strains adhered to both solvents, but they adhered more to toluene than chloroform.

#### 3.2.6. Antagonistic Activity of LAB Against Selected Bacterial Pathogens

All the isolates displayed good antagonistic activity against all the test pathogens, except *P. pentosaceus* A052M, which did not show any inhibition of *S. aureus* and produced small inhibition zones against *E. coli* and *L. monocytogenes*. (Table 5). Some isolates showed substantial statistically significant differences (*p* < 0.05) in their ability to inhibit the selected pathogens. *E. durans* OR794489.1 was the most potent against *E. coli* (21 mm) and *S. aureus* (17 mm), while *L*. *monocytogenes* was mostly inhibited by *P. pentosaceus* A056M and *E. faecium* A04M. The LAB strain that inhibited the growth of *S*. *aureus* the most was *L*. *citreum* A102M. Overall, *P. pentosaceus* A052M was the least inhibitory to the pathogens, while E. *durans* A05M was the most potent.

### 3.3. Antifungal Activity of Viable LAB and Their Cell-Free Supernatants

Fungi isolated from the maize samples were identified as *Alternaria tenuissima* F22F, *Fusarium oxysporum* F44F, *Penicillium corylophilum* F45F, and *Cladosporium halotolerans* F46F. *A. tenuissima* and *F. oxysporum*, known to produce mycotoxins, were chosen as test fungi against which the antifungal properties of LAB were screened.

**Figure 6:**
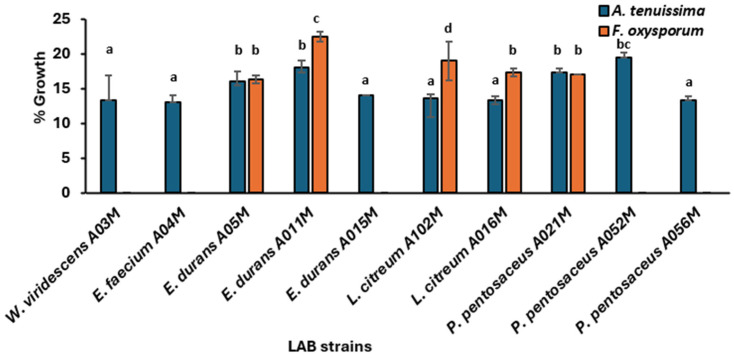
Antifungal activity of LAB viable culture against *A. tenuissima*, *F. oxysporum* and *A. flavus.* Data are represented as the mean (n = 3); error bars indicate standard deviations. Superscripts in lower case present the significant difference at *p* < 0.05.

The ten LAB strains were evaluated for antifungal activity against *Alternaria tenuissima* F22F and *Fusarium oxysporum* F44F. These strains displayed diverse levels of fungal growth inhibition (Figure 6). *A. tenuissima* F22F was the most sensitive strain, and its growth was restricted by all LAB viable cells (Figure 6). *W. viridescens* A03M, *E. faecium* A04M, *E. durans* A015M, and *P. pentosaceus* A052M and *P. pentosaceus* A056M showed no inhibitory activity against *F. oxysporum* F44F. *E. durans* A011M was the most effective against *F. oxysporum*, while *P. pentosaceus* A052M was the most effective against *A. tenuissima*.

**Figure 7:**
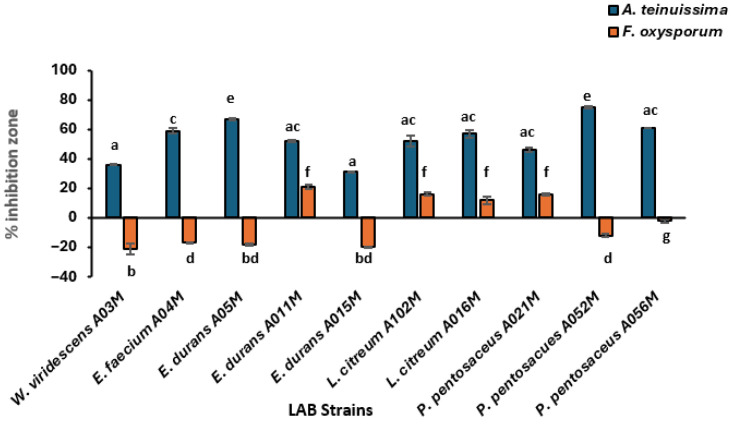
Antifungal activity of cell-free supernatants of LAB isolates against *A. tenuissima* and *F. oxysporum.* Data are represented as the mean (n = 3); error bars indicate standard deviations. Superscripts small case present the significant difference at *p* < 0.05.

The antifungal activity of CFS of LAB strains was assessed using the mould agar spot assay. As was observed for viable LAB cells, their CFSs exhibited varying levels of activity against *Alternaria tenuissima* (Figure 7). The CFS of *E. durans* and *P. pentosaceus* A052M showed high antifungal activity against *A*. *tenuissima.*

Cell-free supernatants of *E. faecium* A04M, *W. viridescens* A03M, *E. durans* A05M, and *P. pentosaceus* A052M and A056M strains exhibited no inhibitory activity against *F. oxysporum* F44F. The ability of some CFSs of LAB strains (*E. durans* A011M and *L. citreum* A102M) to inhibit the growth of *A. teinuissima* and *F. oxysporum* was not significantly different (*p* > 0.05), while others (*P. pentosaceus* and *W. viridescens*) showed significant differences (*p* < 0.05) across the trials. The most effective CFSs against *F. oxysporum* were those of *W. viridescens* A03M and *E. durans* A015M, while *E. durans* A011M was the least efficient. Then, for *A. teinuissima*, the most effective CFS was that of *E. durans* A015M, while the least effective was that of *P. pentosaceus* A052M. Overall, the CFS of LAB inhibited the growth of *F. oxysporum* more than that of *A*. *teinuissima.* However, both the viable strains and CFSs of *E. durans* A011M, *L. citreum* A102M, *L. citreum* A016M, and *P. pentosaceus* A021M showed inhibitory effects against both fungi.

## 4. Discussion

The capability of probiotics to confer beneficial health effects to the host depends on their administration in adequate proportions, as well as their survival both in products and during gastrointestinal transit [32]. Maize-derived LAB isolates were identified using 16S rDNA sequencing, which is considered the gold standard for identifying anaerobic bacteria. The morphological features of the LAB isolates were consistent with molecular characterization. *Enterococcus* sp., *Pediococcus* sp., and *Leuconostoc* sp. were all cocci-shaped. For *Weissella* sp., both single and short-pair rods were observed. This is consistent with a description by Björkroth et al. [33] of *W. viridescens*, which is characterised as slightly irregular rods with rounded to tampered ends that occur singly or in pairs.

Based on 16S rDNA gene sequencing, suggested by García-Hernández et al. [34] as a specific, quick, and accurate approach for identifying LAB, a diverse range of LAB species was identified, and their distribution did not show any significant trend among the samples. Sakandar et al. [35] reported that the 16S rDNA sequencing confirmed the identification and nomenclature of the LAB. Many researchers have reported the isolates from the species isolated in this study as potential probiotics [7,25,28].

Since it is important for probiotics to survive passage through the upper digestive tract and into the large intestine, potential strains are evaluated for their acid and bile salt tolerance. In our study, only *E. durans* survived a three-hour incubation at pH 1.5. Similarly, Soliemani et al. [36] reported that *E. durans* thrived in acidic environments, with growth ranging from 86.23% to 114% at pH levels 2 and 3. The high frequency of enterococci in processed foods reflects their ability to withstand high temperatures and harsh environmental conditions, making them potential markers of the sanitary quality of food [37]. These traits of enterococci are among the most important elements determining their survival.

At pH 2.0, no growth or sparsity was recorded among the isolates, which is consistent with the findings of Padmavathi et al. [25] who reported that LAB isolates had low to moderate tolerance at pH 2. However, *Pediococcus*, *Leuconostoc*, and *Weissella* sp. are among the isolates that can withstand pH levels of 2.5 and 3. The capacity of the isolates to survive in acidic environments displayed species or strain specificity. The resistance mechanism of LAB towards low pH is strain- or species-dependent, and involves the specific metabolites and/or proteins produced by the organisms [38]. Similar results to our study were reported by Abushelaibi et al. [7] and Le and Yang [39] for LAB isolated from camel milk and traditional Korean salted squid. The isolates demonstrated the ability to survive exposure to high acidic environments (pH 2, pH 3), thus indicating sustained viability at both pH levels. This indicates notable levels of resistance to acid stress, suggesting effective physiological adaptation to acidic conditions.

A probiotic must also be able to withstand the effects of harmful metabolites such as phenols released during the digestion of food [26]. Phenol is a microbial toxin produced in the gastrointestinal tract (GIT) by the deamination of certain amino acids derived from protein digestion [39]. Phenol has bacteriostatic properties and inhibits LAB growth; hence phenol resistance is an important probiotic attribute [40]. The LAB isolates in this study exhibited high resistance to different phenol concentrations, consistent with findings reported by Panda et al. [40], who observed comparatively good survival rates of LAB, ranging from 0.2% up to 90%, and the relative growth of 50% at a phenol concentration of 0.4%. In contrast, *Lactobacillus* sp. G3_4_1TO_2_ displayed a very low tolerance (2%) to 0.2% phenol concentration. *Lactobacillus* sp. G3_4_1TO_2_ and *Lactobacillus fermentum* exhibited low cell surface hydrophobicity (10–15%) [25] while the isolates in the present study exhibited markedly higher adhesion capabilities, reaching up to 90%.

Bile tolerance is regarded as an important characteristic of LAB strains, allowing them to survive, develop, and function in the gastrointestinal tract [26]. Bile is involved in both specific and general gut defense systems, and the extent of its inhibitory activity is mostly determined by bile salt concentrations.

Physiological ox gall concentration for bile salt tolerance has been recommended as 0.3–0.5% [40]. Accordingly, the tolerance of *Pediococcus*, *Enterococcus*, *Leuconostoc*, and *Weissella* was observed upon their exposure to 0.3 and 0.5% concentrations, and reports have revealed that these LAB from food samples are bile-tolerant. At 0.3%, the growth of all isolates was reduced after 6 h of incubation, but at least 100% of the isolates survived, though variably. This is consistent with various results from researchers who reported the slowed and reduced growth of potential probiotics in the presence of bile salts [40,41]. The strains remained viable even at higher bile salt concentrations.

Antibiotic resistance has evolved so widely among pathogenic microorganisms that there are concerns that it may occur in beneficial microbes. Evaluating the antibiotic resistance profile pattern of isolates is crucial to limit the use of probiotic cultures with transferable antibiotic resistance genes. All tested LAB were resistant to oxacillin, aligning with previous studies by Yerlikaya et al. [28]. Jafari-Nasab et al. [42] also found high sensitivity of LAB to various antibiotics but resistance to kanamycin and oxacillin. According to Neut et al. [43], probiotics are resistant to oxacillin and metronidazole due to their narrow spectrum of activity and so have limited effects on the microbiota.

Generally, many probiotics are susceptible to antibiotics that inhibit protein synthesis, such as chloramphenicol, erythromycin, and tetracycline [44,45]. The isolates in this investigation were all susceptible to chloramphenicol, and 90% were susceptible to erythromycin and tetracycline. Abriouel et al. [44] discovered that *Lactobacillus* and *Enterococcus* strains are susceptible to chloramphenicol, erythromycin, and tetracycline, which is consistent with our findings. The LAB tested against streptomycin were categorised as resistant based on the cut-off values described by Yerlikaya et al. [28]. Lactic acid bacteria are frequently found to be resistant to the class of aminoglycosides to which streptomycin belongs. One of the most important factors in aminoglycoside resistance is aminoglycoside-modifying enzymes (AMEs) [46]. The mechanisms usually include antibiotic inactivation by enzymes such as phosphotransferase, acetyltransferase, and nucleotidyltransferase [45]. Active electron transport is required for aminoglycoside uptake into cells; therefore, this class of antibiotics inherently lacks activity against anaerobic bacteria [47,48]. AMEs can affect their affinity for targets, reducing their efficacy. Aminoglycosides synergize with other antibacterial classes, increasing bacterial resistance [48]. Streptomycin resistance may be genetic, potentially transferred laterally in the gastrointestinal tract. Therefore, it’s not recommended to use probiotics while taking streptomycin.

The study analysed the cell surface hydrophobicity of isolates by observing their ability to adhere to toluene and chloroform (Figure 5) using the MATH method. This hydrophobicity determines the hydrophilic nature of the bacterial cell surface and membrane, which is linked to the strain’s ability to attach to nonpolar sources [49,50]. Probiotic bacteria use cell hydrophobicity to colonize the gastrointestinal tract, preventing enteropathogen binding. The bacteria must survive in the gastrointestinal system and stick to the colon mucosa to protect against pathogens [51]. This highlights the importance of cell surface hydrophobicity in probiotic bacteria.

The comparatively high hydrophobicity obtained indicated that the isolates have good binding properties and should exert significant barrier function in the host. Based on the degree of adhesion to hydrocarbons, Sánchez-Ortiz et al. [52] categorized bacterial strains into three categories, including under 30% (<30%) as low hydrophobic, between 30 and 60% (≥30 and <60%) as moderately hydrophobic, and above 60% (≥60%) as strongly hydrophobic. In accordance with the above classification, 90% of the analyzed organisms were strongly hydrophobic to toluene, while 60% were strongly hydrophobic to chloroform. Different studies reported different adherence percentages of potential probiotic organisms, including *Pediococcus*, *Enterococcus*, *Leuconostoc*, and *Weissella* species [28,50,53,54,55]. All the *Pediococcus* species were moderately hydrophobic, while ≥95% hydrophobicity was recorded for all enterococcus species. This is consistent with the report by Nascimento et al. [53], who recorded 100% hydrophobicity by *Enterococcus* sp. The hydrophobic differences between the potential probiotics may result in variability in their colonising ability. Therefore, strains with high cell hydrophobicity may have easier access to soluble materials and organic matter associated with the intestinal mucosa [54]. Hydrophobic components found in an organism’s outer membranes generate cell surface hydrophobicity [50]. Probiotic bacteria use cell hydrophobicity to attach to the intestinal epithelium and colonize the gastrointestinal tract, giving benefits such as preventing the binding of the enteropathogens to these sites. Probiotic bacteria must be able to survive in the gastrointestinal system and stick to the colon mucosa to protect against pathogens [51].

Another key characteristic to look out for in a probiotic is its antagonistic activity against pathogens. The LAB isolates demonstrated antibacterial efficacy against the selected bacterial pathogens, which are frequently linked with gastrointestinal illness. Their antimicrobial properties may be attributed to metabolites produced during LAB growth, such as bacteriocins, which may play a significant role in the functionality of probiotics [56,57]. *Pediococcus pentosaceus* ST65ACCC, isolated from Brazilian artisanal cheese, can inhibit *Listeria monocytogenes* [58]. *Lactobacillus* species are well-studied and recognized probiotics. *Lactobacillus acidophilus* CM1 and *L. delbrueckii* OS1 isolated from dietary sources such as curd, milk and pickles, were previously reported to inhibit *E. coli* and *S. aureus*, with inhibition zones of 11 mm and 12 mm, respectively [59]. In comparison, the inhibition zone recorded for our isolates ranged between 13 to 21 mm against *E. coli* and 17 mm against *S. aureus*. These results suggest that our isolates have exhibit stronger antimicrobial activity than some commonly reported *Lactobacillus* species.

Fungi are one of the major crop pathogens, and an enormous quantity of agricultural products is lost annually due to the presence of these organisms in and on the crops. The most prevalent spoilage fungi in agricultural produce include *Aspergillus*, *Alternaria*, *Penicillium*, and *Fusarium* species [60,61]. These genera can produce mycotoxins, which are regarded as a significant health danger. Therefore, two toxigenic fungal species were subjected to LAB activity, and results showed that some of the LAB isolates have strong antifungal activity and bio-preservation potential. Similar to our findings, Fugaban et al. [11] discovered that *Pediococcus pentosaceus* and *P. acidilactici* from silage inhibit the growth of mycotoxigenic fungal including *Alternaria alternate, Aspergillus niger*, *Penicillium chrysogenum*, *Cladosporium sphaerospermum*, *and P. expansum*. Saladino et al. [62] demonstrated a similar result on the antifungal capabilities of LAB against mycotoxigenic fungi, utilising an agar diffusion method. The CFS derived from LAB (*P. acidilactici* JY03, *W. paramesenteroides* JT13 and *Lactobacillus sucicola* JT03) inhibit the growth of *P. digitatum* in 48 h [59]. According to Karami et al. [63], *L. alimentarius* exhibited antifungal activity, with inhibition zone diameters of up to 10 mm and 12 mm against *P. notatum* and *A. fulvous*, respectively. Similarly, *L. delbrueckii* sp. showed inhibition zones of 11 mm against *A. fulvous* and 13 mm against *P. notatum*. In contrast, in the present study, *E. durans* produced inhibition zones of up to 22.5 mm diameter against the tested fungi, depicting stronger antifungal activity than that previously reported for other LAB.

In addition to activity against pathogenic bacteria, *E. durans*, *L. citreum*, and *P. pentosaceus* showed high antifungal activity. This may be due to their ability to produce a range of antifungal metabolites, including organic acids, low-molecular-weight compounds, and even enzymes [61]. Although this study did not directly investigate the mechanisms underlying the functional traits of the LAB strains, these traits may be mediated by coordinated physiological adaptations, including stress response systems, bacteriocin production, membrane modifications, and metabolic flexibility, which collectively enhance survival and functional performance in acidic and competitive environments. Acid and bile tolerance may be associated with maintenance of intracellular pH through proton extrusion systems and alteration of cell membrane composition. Similarly, the antimicrobial activity may be due to the production of short-chain fatty acids, hydrogen peroxide, bacteriocins or other antimicrobial peptides. These potential mechanisms may contribute to the observed functional properties and, therefore, warrant further investigation.

The use of food-associated LAB as biopreservatives represents a viable alternative to chemical preservatives due to their generally recognized as safe (GRAS) status. Moreover, the application of LAB-derived enzymes for mycotoxin degradation and decontamination may provide significant health benefits, as they have the potential to inhibit the growth of toxigenic fungi.

## 5. Conclusions

This study examined maize samples to identify novel probiotics. We assessed their intestinal adaptation, antibiotic resistance, and their inhibitory activity against pathogenic bacteria. Although this study was conducted in vitro, all the LAB strains identified show promising probiotic potential and could be utilised as biocontrol against pathogens and toxigenic fungi in the food sector, with *E. durans* demonstrating particularly remarkable properties. All the strains demonstrated resistance to low pH, phenol, and tolerance to bile salt, indicating their ability to survive and potentially colonize the gastrointestinal environment. They also showed cell hydrophobicity and strong antibacterial and antifungal activities against the mycotoxigenic fungi isolated from maize grains. It is therefore suggested that both traditional and industrial foods, especially maize products, can be fortified with these LAB isolates to improve flavour, nutritional quality, and food safety. Further technological characterization of the isolated LAB, including screening these isolates for enzymes involved in fungal growth inhibition and potential mycotoxin degradation, is currently underway as part of our ongoing research.

## Figures and Tables

**Table 1:** Antibiotic cut-off values used for the LAB isolates’ susceptibility.

Antibiotic	Resistance	Moderate	Susceptible
Chloramphenicol	≤13	14–17	≥18
Erythromycin	≤13	14–17	≥18
Fusidic acid	≤15	16–20	≥21
Oxacillin	6–8	9–19	≥20
Penicillin G	6–8	9–19	≥20
Streptomycin	≤12	-	≥13
Tetracycline	≤14	15–18	≥19

**Table 2:** Identification of LAB isolates using 16S rDNA gene sequencing.

Serial Number	Strain Number	Nomenclature	Similarity (%)	Accession Number
1	A05M	*Enterococcus durans*	100	OR794489.1
2	A011M	*Enterococcus durans*	97	OR567519.1
3	A014M	*Enterococcus durans*	97	
4	A015M	*Enterococcus durans*	96	OR793151.1
5	A04M	*Enterococcus faecium*	96	OR793166.1
6	A102M	*Leuconostoc citreum*	99	OR600361.1
7	A016M	*Leuconostoc citreum*	92	OR794488.1
8	A021M	*Pediococcus pentosaceus*	99	OR643697.1
9	A025M	*Pediococcus pentosaceus*	96	
10	A052M	*Pediococcus pentosaceus*	96	OR794367.1
11	A056M	*Pediococcus pentosaceus*	95	OR591164.1
12	A02M	*Pediococcus pentosaceus*	87	-
13	A07M	*Pediococcus pentosaceus*	92	-
14	A017M	*Pediococcus pentosaceus*	79	-
15	A054M	*Pediococcus pentosaceus*	94	-
16	A03M	*Weissella viridescens*	95	OR569669.1
17	A035M	*Weissella viridescens*	86	-
18	A036M	*Weissella viridescens*	86	-
19	A045M	*Weissella viridescens*	98	-
20	A048M	*Weissella viridescens*	90	-
21	A049M	*Weissella viridescens*	90	-
22	A039M	*Weissella viridescens*	98	-
23	A051M	*Weissella viridescens*	94	-

**Table 3:** Antibiotic susceptibility descriptions of the LAB isolates.

Isolates				Antibiotics				
	C (25 µg)	E (5 µg)	FC (10 µg)	OX (5 µg)	NO (5 µg)	PG (1 Unit)	S (10 µg)	T (25 µg)
W. viridescens A03M	S	R	R	R	R	R	R	S
E. faecium A04M	S	S	MS	R	MS	MS	R	S
E. durans A05M	S	S	R	R	R	MS	R	S
E. durans A011M	S	MS	MS	R	R	MS	R	S
E. durans A015M	S	MS	R	R	MS	MS	R	S
L. citreum A102M	MS	MS	R	R	R	S	R	S
L. citreum A016M	S	S	MS	R	MS	MS	R	S
P. pentosaceus A021M	S	S	R	R	R	MS	R	S
P. pentosaceus A052M	MS	R	R	R	R	R	R	R
P. pentosaceus A056M	S	S	R	R	R	R	R	MS

C—Chloramphenicol, E—Erythromycin, FC—Fusidic acid, Ox—Oxacillin, NO—Novobiocin, P—penicillin G (10 µg), S—Streptomycin, and T—Tetracycline (30 µg). R—Resistance, MS—Moderately susceptible, S—Susceptible.

**Table 4:** Percentage of antibiotic susceptibility of potential probiotic LAB isolates.

Antibiotics	*n* = 10		
	R (%)	MS (%)	S (%)
Chloramphenicol	0	20	80
Erythromycin	20	30	50
Fusidic acid	70	30	0
Oxacillin	100	0	0
Novobiocin	70	30	0
Penicillin G	30	60	10
Streptomycin	100	0	0
Tetracycline	10	10	80

R: Resistant, MS: Moderately susceptible, S: Susceptible, %: Percentages based on 10 LAB isolates tested.

**Table 5:** Antimicrobial activities of LAB isolates against selected bacterial pathogens.

LAB Isolates	Bacterial Pathogens (Mean Diameter of Inhibition Zone (mm ± SD)		
	*E. coli*	*S. aureus*	*L. monocytogenes*
*W. viridescens* OR569669.1	15.0 ± 1.0	14.0 ± 1.5	11.0 ± 0.5
*E. faecium* OR793166.1	15.0 ± 1.5	15.0 ± 3.5	18.0 ± 1.0
*E. durans* OR794489.1	21.0 ± 1.1	17.0 ± 0.5	14.0 ± 1.1
*E. durans* OR567519.1	16.0 ± 1.1	11.0 ± 2.0	14.0 ± 1.5
*L. citreum* OR600361.1	14.7 ± 1.1	16.7 ± 1.5 ^b^	12.0 ± 1.5
*E. durans* OR793151.1	14.7 ± 0.5	11.0 ± 2.0	6.0 ± 2.1
*L. citreum* OR794488.1	18.3 ± 0.5 ^a^	13.0 ± 1.0 ^ab^	14.0 ± 0.5
*P. pentosaceus* OR643697.1	14.3 ± 1.5	14.0 ± 0.0	17.0 ± 0.5 ^bc^
*P. pentosaceus* OR794367.1	8.0 ± 1.4 ^a^	0 ± 0.0	6.0 ± 0.5 ^ac^
*P. pentosaceus* OR591164.1	13.0 ± 0.0	12.5 ± 2.1	18.7 ± 1.5 ^c^

mm—mean of triplicate readings ± standard deviation. The dissimilar superscript alphabets indicate the significant differences.

## Data Availability

The original contributions presented in this study are included in the article. Further inquiries can be directed to the corresponding author.

## Abbreviations

The following abbreviations are used in this manuscript:

AMEs	Aminoglycoside-modifying enzymes
CFS	Cell-free supernatant
CFU	Colony-forming units
GRAS	Generally regarded as safe
LAB	Lactic acid bacteria
rDNA	Ribosomal deoxyribonucleic acid
PBS	Phosphate buffer saline
PDA	Potato dextrose agar
PCR	Polymerase chain reaction
